# Alpha rhythm slowing in temporal lobe epilepsy across scalp EEG and MEG

**DOI:** 10.1093/braincomms/fcae439

**Published:** 2024-12-05

**Authors:** Vytene Janiukstyte, Csaba Kozma, Thomas W Owen, Umair J Chaudhary, Beate Diehl, Louis Lemieux, John S Duncan, Fergus Rugg-Gunn, Jane de Tisi, Yujiang Wang, Peter N Taylor

**Affiliations:** CNNP Lab, Interdisciplinary Computing and Complex BioSystems Group, School of Computing, Newcastle University, NE4 5DG Newcastle upon Tyne, UK; CNNP Lab, Interdisciplinary Computing and Complex BioSystems Group, School of Computing, Newcastle University, NE4 5DG Newcastle upon Tyne, UK; CNNP Lab, Interdisciplinary Computing and Complex BioSystems Group, School of Computing, Newcastle University, NE4 5DG Newcastle upon Tyne, UK; Department of Clinical and Experimental Epilepsy, UCL Queen Square Institute of Neurology, WC1N 3BG London, UK; Department of Clinical and Experimental Epilepsy, UCL Queen Square Institute of Neurology, WC1N 3BG London, UK; Department of Clinical and Experimental Epilepsy, UCL Queen Square Institute of Neurology, WC1N 3BG London, UK; Department of Clinical and Experimental Epilepsy, UCL Queen Square Institute of Neurology, WC1N 3BG London, UK; Department of Clinical and Experimental Epilepsy, UCL Queen Square Institute of Neurology, WC1N 3BG London, UK; Department of Clinical and Experimental Epilepsy, UCL Queen Square Institute of Neurology, WC1N 3BG London, UK; CNNP Lab, Interdisciplinary Computing and Complex BioSystems Group, School of Computing, Newcastle University, NE4 5DG Newcastle upon Tyne, UK; Department of Clinical and Experimental Epilepsy, UCL Queen Square Institute of Neurology, WC1N 3BG London, UK; Faculty of Medical Sciences, Newcastle University, NE2 4HH Newcastle upon Tyne, UK; CNNP Lab, Interdisciplinary Computing and Complex BioSystems Group, School of Computing, Newcastle University, NE4 5DG Newcastle upon Tyne, UK; Department of Clinical and Experimental Epilepsy, UCL Queen Square Institute of Neurology, WC1N 3BG London, UK; Faculty of Medical Sciences, Newcastle University, NE2 4HH Newcastle upon Tyne, UK

**Keywords:** scalp EEG, MEG, alpha rhythm, alpha slowing, temporal lobe epilepsy

## Abstract

EEG slowing is reported in various neurological disorders including Alzheimer’s, Parkinson’s and Epilepsy. Here, we investigate alpha rhythm slowing in individuals with refractory temporal lobe epilepsy compared with healthy controls, using scalp EEG and magnetoencephalography. We retrospectively analysed data from 17 (46) healthy controls and 22 (24) individuals with temporal lobe epilepsy who underwent scalp EEG and magnetoencephalography recordings as part of presurgical evaluation. Resting-state, eyes-closed recordings were source reconstructed using the standardized low-resolution brain electrographic tomography method. We extracted slow 6–9 Hz and fast 10–11 Hz alpha relative band power and calculated the alpha power ratio by dividing slow alpha by fast alpha. This ratio was computed for all brain regions in all individuals. Alpha oscillations were slower in individuals with temporal lobe epilepsy than controls (*P*<0.05). This effect was present in both the ipsilateral and contralateral hemispheres and across widespread brain regions. Alpha slowing in temporal lobe epilepsy was found in both EEG and magnetoencephalography recordings. We interpret greater slow alpha as greater deviation from health.

## Teaser

This study investigates alpha rhythm slowing in individuals with epilepsy compared with healthy controls, in EEG and magnetoencephalography (MEG). Janiukstyte *et al*. report that alpha oscillations are slower in individuals with epilepsy than controls (*P* < 0.05), seen in both the ipsilateral and contralateral hemispheres and across widespread brain regions.

## Graphical Abstract

**Graphical Abstract:**
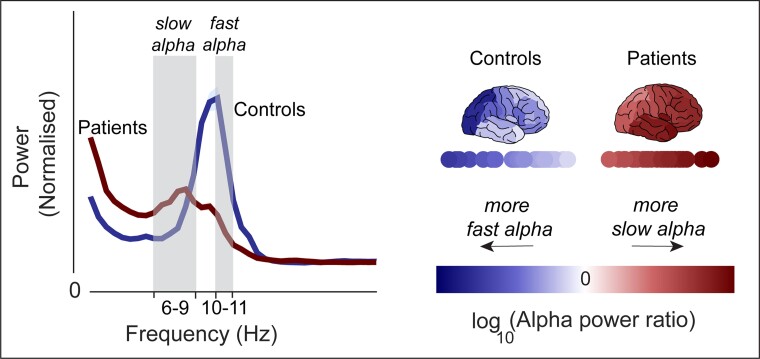

## Introduction

The alpha rhythm 8–13 Hz^1-5^ is a dominant neurophysiological oscillation in the occipital and parietal regions,^1,4,6^ during a relaxed, eyes-closed, wakeful state observed in most humans.^1,2,4^ It is believed to be generated by corticothalamic connections^7^ and is alerted by the following: attention,^1-3,8,9^ language,^3^ sensory processing^1^ and working memory.^2,3,9^ Additionally, alpha may be involved in inhibitory mechanisms and suppression, which reduce cortical excitability in non-essential cortical regions at present^2,3,5,8-10^ Deficiency of the latter functions may alter the alpha rhythm and facilitate pathological activity^11-15^ Therefore, alpha rhythm alterations may indicate early brain health degradation.

EEG slowing is a hallmark of neurodegenerative, metabolic and neuropsychiatric diseases,^3,16-23^ which can be difficult to distinguish from healthy aging.^21^ Alpha rhythm slowing has also been found in epilepsy, showing reduced alpha power and topographical shift of maximum relative power to more frontal sites.^19,20^ Recent observations supports alpha rhythm alterations^24,25^ and reports reduced peak alpha frequency in individuals with mesial temporal lobe epilepsy (TLE) and their asymptomatic relatives in EEG^26^ and magnetoencephalography (MEG).^27,28^ Overall, alpha slowing in epilepsy remains under-investigated and its clinical significance is unknown.

The heterogeneity of alpha rhythm slowing across various diseases and their syndromes is usually considered a physiological by-product. Specifically, the role of corticothalamic connections, cortical neural networks and thalamic influences on alpha oscillations are heavily debated,^1,2,7,9,15,17,29^ with studies supporting the idea that alpha oscillations are generated, maintained or propagated via multiple networks. Alternatively, the alpha rhythm is thought to be involved in the inhibitory mechanism, which is mediated by GABA interneurons. In this hypothesis, compromised GABA interneurons facilitate neuronal excitation, which relates to seizure generation^30,31^ and is linked to Alzheimer’s disease neuropathology.^3,32-34^ Multiple genetic mutations associated with epilepsy syndromes^26,30,31,35^ and antiseizure medication may also contribute to alpha power alterations.^26,36^ Overall, a slower alpha rhythm is associated with behaviour symptoms that are common across multiple neurological diseases.

Here, we extend current literature, by extracting relative band power from scalp EEG and MEG individual adult cohorts. We also present the spatial representation of alpha power across hemispheres in left and right TLE. First, we quantified the distribution of alpha power by computing slow and fast alpha power in resting-state scalp EEG and MEG recordings, in healthy controls and individuals with TLE. Initially, we relate the loss of normalized relative band power in scalp EEG and MEG recordings to previously published literature.^24,27^ Next, we investigate the spatial patterns of alpha power in individuals with TLE, compared with healthy controls.

## Materials and methods

### Scalp EEG and MEG subjects

EEG data were acquired from 17 healthy volunteers and 22 individuals with drug-resistant TLE undergoing presurgical evaluation at the National Hospital for Neurology and Neurosurgery (NHNN; part of the UCLH National Health Service Foundation Trust, Queen Square, London, UK) were recruited. The patients had no previous neurosurgery or invasive neurosurgical procedures prior to the EEG recordings, which were collected for research purposes during pre-surgical assessment. These data have been described previously.^12^

MEG data were collected from 46 healthy volunteers and 24 individuals with TLE who were undergoing pre-surgical evaluation.

There were no significant differences present between control and patient cohorts based on age and sex (Table 1). Of the 37 patients across EEG and MEG, 1 had diabetes, 2 had intellectual disability, 3 had hypertension, 3 had other (lupus/hyperthyroidism), 13 had neuropsychiatry related comorbidities (mild or severe depression, anxiety, aggression, ADHD, postictal psychosis) and 19 had no comorbidities noted. There was no significant difference in alpha power in those with and without comorbidities. A summary of EEG and MEG cohorts is available in Supplementary Tables S1–S4.

**Table 1:** Descriptive statistics of healthy controls and patients in scalp EEG and MEG cohorts

	Scalp EEG			MEG		
	Controls	Patients	Test statistic	Controls	Patients	Test statistic
Number of subjects	17	22	N/A	46	24	N/A
Age (y): mean (SD)	31.9 (6.46)	34.2 (10.1)	*P* = 0.41, es = 8.69	30.5 (6.7)	33.9 (9.5)	*P* = 0.08, es = −1.75
Sex: male/female (%)	11/6 (65%)	9/13 (41%)	*P* = 0.14, χ^2^ = 2.17	16/30 (35%)	12/12 (50%)	*P* = 0.48, χ^2^ = 1.52
Epilepsy lateralisation: Left/right (%)	N/A	14/8 (64%)	N/A	N/A	9/15 (36%)	N/A
Age of epilepsy onset (y): mean (SD)	N/A	12.9 (8.3)	N/A	N/A	14.2 (7.7)	N/A
Duration of epilepsy (y): mean (SD)	N/A	21.3 (13.2)	N/A	N/A	19.8 (11.8)	N/A

### Scalp EEG recording and processing

Eyes-closed resting-state EEG data were recorded from 30 scalp electrodes using a commercial MR-compatible system (BrainAmp MR and Vision Analyzer) with a sampling rate of 5000 Hz using a common average reference, positioned according to the 10–20 internation system.^37^ All EEG data were acquired at the MRI Unit of the Epilepsy Society (Chalfont St Peter, Buckinghamshire, UK). Ocular and cardiac activities were captured using two reference electrodes: EOG and ECG. The first 30 s of the recordings were disregarded to allow subjects to settle.

The EEG recordings were processed in MATLAB and Brainstorm, using previously described methods,^12^ as follows: EEG recordings were downsampled to 250 Hz. We used ICBM152 anatomical MRI template in standard space and the boundary element method to create a realistic head model template. Digitized Brainstorm electrode locations were co-registered to the template, projecting electrodes perpendicularly to the nearest anatomical scalp location and manually reviewed to confirm satisfactory warping. We bandpass filtered the sensor time series between 1 and 47.5 Hz. We manually inspected the individual interictal recordings for artefacts, spikes or potential pathological events. Signal space projection was performed to identify artefactual components (ECG heartbeat artefact) with manual intervention when necessary. Ocular artefacts could not be accurately identified and were therefore retained to preserve biological signal, particularly given that eyes-closed recordings were acquired.

The resulting data were then source reconstructed using the standardized low-resolution brain electrographic tomography method with a realistic head model derived from boundary element method and spatially downsampled based on Lausanne parcellation, into 68 regions of interest (ROIs).^38^ The most artefact-free 60-s epoch segments were selected from each subject to represent normative power EEG baselines.

Note that in low-resolution parcellation, some ROIs represent large neocortical areas and summate activity from multiple neighbouring locations. Constrained manifestation of source mapping orients the overlapping current magnitudes from contradictory sides of the sulci in opposite directions. To counteract these issues, the source recordings were sign-flipped and subsequently averaged to create a single time series per region.

### MEG recording and processing

Eyes-closed resting-state MEG recordings were acquired using two 275-channel CTF whole-head MEG systems, at different locations. The healthy control cohort data were collected at CUBRIC, Cardiff, UK (as part of the MEG UK partnership), and the patients were recruited at NHNN and the data recorded at the Wellcome Centre for Human Neuroimaging of the UCL Queen Square Institute of Neurology, London, UK. The MEG data were processed in Brainstorm using previously described methods as follows.^13^ In summary, MEG sensor locations were coregistered to the individual’s MRI scans using fiducial markers and manually reviewed to confirm satisfactory alignment. MEG data were downsampled to 600 Hz. Independent component analysis was used to aid manually removing ocular and cardiac artefacts. Artefact-free MEG recordings were source reconstructed using minimum-norm imaging technique standardized low-resolution brain electrographic tomography^39^ and an overlapping spheres head model. Similar to EEG (see previous section), the time-series were spatially downsampled into 68 ROIs. Finally, the most artefact-free 70-second segments were extracted to produce normative power MEG baselines.

### Computing relative scalp EEG and MEG power

**Figure 1:**
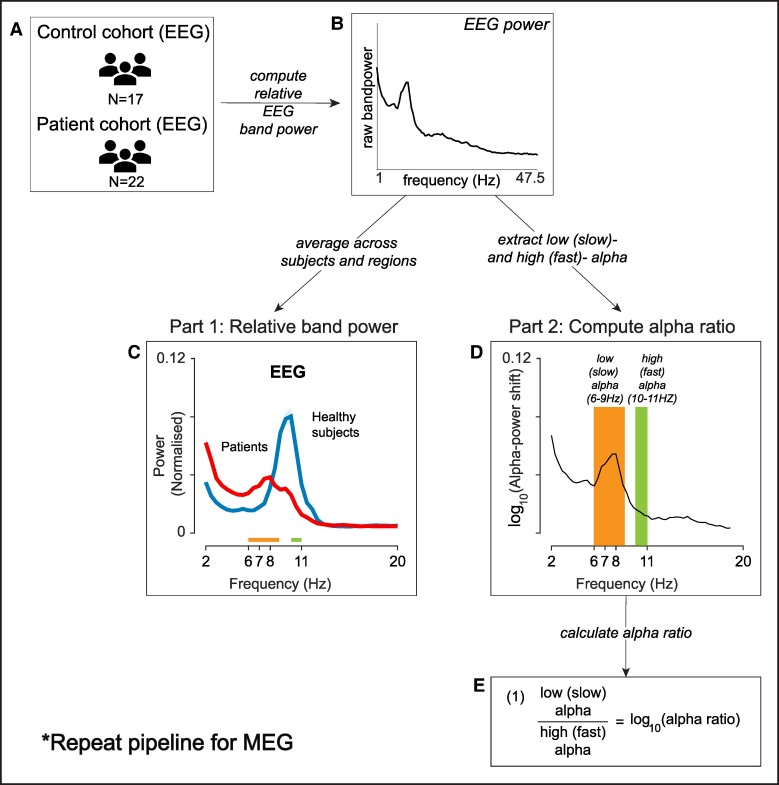
**Processing pipeline to calculate relative band power and to compute alpha power ratio in scalp EEG and MEG.** (**A**) EEG recordings were collected for healthy controls and individuals with TLE cohorts. (**B**) We computed relative band power across individual subjects, for EEG recordings. (**C**) Relative band power was averaged across subjects and 68 ROIs and normalized. The resulting normalized power from 17 controls was plotted against 22 individuals with TLE, for EEG. (**D**) For each subject, we also extracted the slow 6–9 Hz and fast 10–11 Hz alpha relative band power, as described in study by Abela *et al*.^24^ (**E**) The ‘alpha power log ratio’ was then calculated as the ratio of slow over fast alpha power. *The processing pipeline is repeated for MEG data.

To construct power spectral densities illustrated in Fig. 1B, we used the Welch’s method using a 2-second sliding window with 1-second overlap in each neocortical region. We then normalized the power spectral density to sum to one, thus computing relative power. This was performed for each subject for each modality (i.e. EEG and MEG control and patient cohorts; Fig. 1A and B).

### Plotting relative band power

To illustrate the alpha power shift, a measure of global band power was defined as the band power averaged across individuals and the 68 ROIs. We plotted the global band power for the healthy controls and patient cohorts band-wise between 1 and 20 Hz, for EEG and MEG independently (Fig. 1C).

### Calculating alpha power log ratio

Our study closely followed the methods and aims described in Abela *et al.*^24^ and Kudo *et al.*^27^ and had three aims: firstly, to validate the reported maximum alpha power shift from faster to slower frequencies, as previously reported.^19,20,24,27^ Second, to investigate whether this effect was driven by hemispheric abnormality in TLE, and third, to evaluate whether the alpha power ratio driven by the slower frequencies feature was spatially widespread across the cortex.

To test our hypotheses, we focused on the slow alpha 6–9 Hz and fast alpha 10–11 Hz, as reported previously.^24,40^ We defined our outcome variable as the ‘alpha-power ratio’ by dividing slow alpha by fast alpha and taking the log function (Fig. 1D). The ratio expression simplifies the interpretation of the alpha power ratio. We interpret high ratio score as having more slow alpha frequency, and we would expect the subject to deviate away from health.

### Statistical analysis and data visualization

We used the Anderson–Darling and Lilliefors tests to check the significance of deviations from normality in the EEG and MEG data. To compare the alpha power ratio across subject cohorts we used unpaired *t*-tests. Similarly, we used paired *t*-tests to check whether spatially global alpha power ratios in controls versus patients were significant. Additional statistical tests with Bonferroni corrections are available in Supplementary Tables S5–S8, S13 and S14. Visualization of the results was performed using the brainPlot^41^ package implemented in MATLAB.

### Ethical approval

All methods were carried out in accordance with relevant guidelines and regulations, and all experimental protocols were approved by Newcastle University Ethics Committee (1804/2020).

## Results

**Figure 2:**
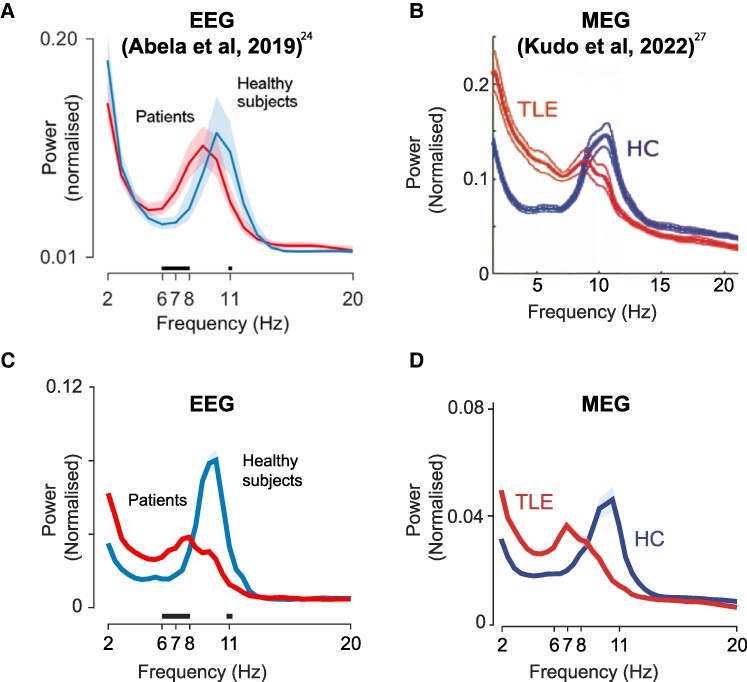
**Loss of fast alpha in EEG and MEG.** Maximum alpha power peak is shifted towards lower-frequencies and reduced in individuals with TLE compared with healthy controls (HCs). Solid lines indicate the power at different frequency intervals and shaded areas illustrate 95% confidence intervals (CI). (**A** and **C**) The alpha power change in EEG and (**B** and **D**) show the alpha power change in MEG. The two plots in the upper row (**A** and **B**) illustrate previously published literature.^24,27^ For visual aesthetics, colour and band power symbols were removed in **B**. (see original in Kudo *et al.*^27^). Both figures used with permission.

**Figure 3:**
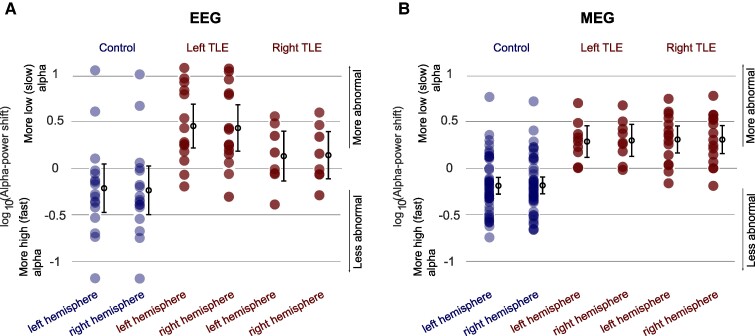
**Alpha power ratio in individual subjects in (A) EEG and (B) MEG.** Illustrating the difference in alpha power ratio between individual healthy controls and individuals with left and right TLE, averaged across regions within a single hemisphere. Each data point is a (log-transformed) alpha power ratio, representing an individual subject. A positive alpha power ratio indicates more slow alpha, associated with greater deviation from healthy subjects. Dots and error bars indicate 95% CI. Unpaired *t*-tests results are provided in Supplementary Tables S5–S8. Note the sample sizes for healthy control, left and right TLE patient cohorts in scalp EEG and MEG: 17 (46), 14 (9) and 8 (15) (per hemisphere).

**Figure 4:**
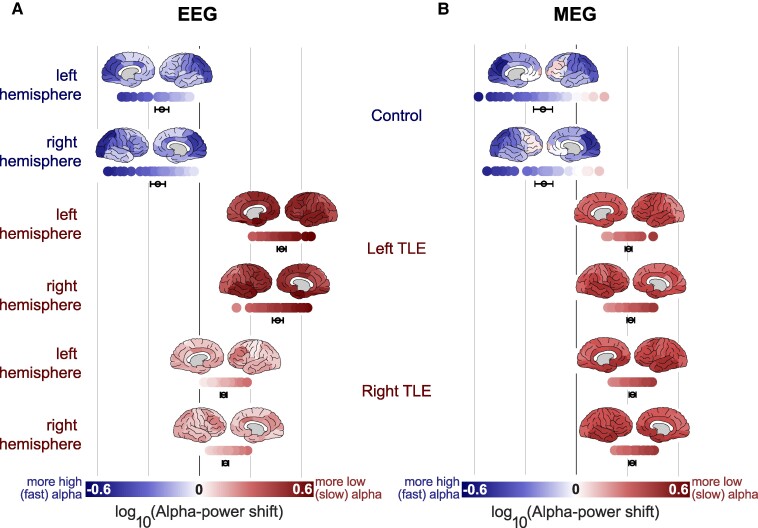
**Loss of fast alpha is spatially global across patients.** Alpha power ratio varies spatially across the cortex in left and right TLE patients compared to healthy controls. Each data point is a (log-transformed) alpha ratio, representing a ROI. There are 34 ROIs in each hemisphere. A positive (red) alpha ratio indicates slow alpha, associated with greater deviation from health. Dots and error bars indicate 95% CI. (**A** and **B**) In the healthy control cohort, fast alpha is maximal in occipital and parietal regions, in EEG and MEG. Slow alpha is maximal anteriorly and in the temporal regions, across most left and right TLE cohorts, in both modalities. Paired *t-*tests results are provided in Supplementary Tables S9–S12.

Alpha rhythm slowing in TLE in scalp EEG and MEG recordings replicates existing literature, shown in Fig. 2.^24,27^ We also investigated the alpha power ratio in individual patients (Fig. 3) and spatially, across hemispheres (Fig. 4). Each data point (alpha power ratio) is calculated by dividing slow alpha by fast alpha power and taking the log function.

### Alpha power ratio

A shift of maximum normalized alpha power peak to the lower frequencies in EEG and MEG was observed in individuals with TLE compared with healthy controls, in agreement with previous findings (Fig. 2A and B).

### Alpha power ratio in individual subjects

To quantify the previous observations, we compared data from individual subjects. Loss of fast alpha frequency was observed in patients with left TLE, in the EEG of both hemispheres, and independent of focus laterality and in both hemispheres in the MEG data (Fig. 3). Anderson–Darling and Lilliefors test of normality confirmed normality in EEG and MEG data.

In EEG, we found a significant bilateral alpha power ratio difference between control and left TLE patient group. Similarly, in MEG, we found significant bilateral alpha power ratio difference between control groups and left and right TLE patient groups. The sample sizes for healthy control, left and right TLE patient cohorts in scalp EEG are 17, 14 and 8, and in MEG: 46, 9 and 15 (per hemisphere). Additional statistical results are available in Supplementary Tables S5–S8.

### Spatial representation of alpha power ratio across subjects in EEG and MEG modalities

Alpha power ratio brain maps are presented in Fig. 4. We report maximal slow alpha in the anterior and temporal regions across most patient cohorts and in both modalities. The right hemisphere, in individuals with right TLE also shows slow alpha spread in the occipital and parietal regions.

To quantify the spatial alpha ratio between groups, we used paired *t*-tests, averaging the alpha power ratio across subjects, with each hemisphere comprising of 34 regions. After Bonferroni’s correction, scalp EEG showed a high fast alpha decrease across both patient hemispheres compared to controls, with more substantial differences in left TLE (*P* = 0.00, tstat = −53.66 to −59.90). In MEG, there was a uniform loss of fast alpha across all TLE patient cohorts, compared to controls (statistical results are in Supplementary Tables S9–S12). Full effect sizes are given in Supplementary Tables S13 and S14. The effect of threshold for high/low alpha is also presented in Supplementary Figs. S1 and S2, and the effect of medication in Supplementary Fig. S3. Similar findings were observed for both effects.

### Estimating the performance of mean alpha power ratio as a potential biomarker

In the following analysis, we investigate the performance of the mean alpha power ratio metric as a biomarker to predict individuals with TLE compared with healthy controls, using area under curve analysis. We hypothesized that individuals with TLE have a more slow alpha than healthy controls.

**Figure 5:**
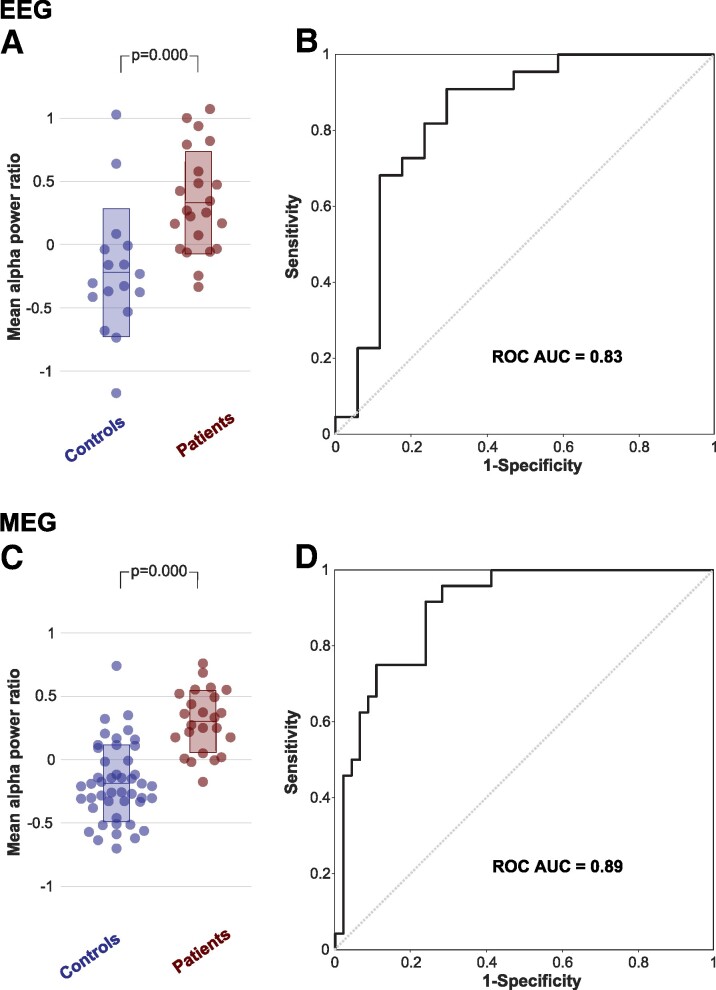
**Performance of mean alpha power ratio as a biomarker.** (A and C) Univariate scatter plot represents mean alpha power ratio across 68 ROIs in each subject. Patients have significantly more slow alpha (*P* = 0.000, unpaired *t*-test) in EEG and MEG. (**B** and **D**) ROC curve, illustrates the performance of the mean alpha power ratio as a biomarker using classification thresholds.

To quantify the performance of mean alpha power ratio as a biomarker, we averaged the alpha power ratio across 68 ROIs and plotted the mean value per subject in Fig. 5A and C. Positive mean alpha ratio indicates a more slow alpha. There was more slow alpha in individuals with TLE in EEG and MEG (*P* = 0.000). Area under curve analysis shown in Fig. 5B and D gave an area under curve of 0.8316 and 0.8995 for EEG and MEG, respectively.

## Discussion

The maximum alpha power peak is shifted towards the lower frequencies in the TLE groups, across scalp EEG and MEG modalities, aligning with previous literature. Additionally, we illustrate the maximum alpha power change from occipital to frontal regions. Taken together, our findings indicate that alpha rhythm slowing was found in individuals with TLE, specifically evident in the occipital and parietal regions where the higher frequency alpha predominates.

While a single routine EEG has <50% sensitivity to capture abnormal EEG waveforms,^42^ repeating routine EEGs increases detection of abnormality to over 90%.^43,44^ We reaffirm the alpha power change to the lower-frequencies in TLE, supporting previous literature^19,20,24,26-28^. We also demonstrate bilateral and widespread alpha slowing in TLE compared to controls, with a more pronounced effect seen in the left TLE on scalp EEG. While previous studies investigated lateralization in EEG^12^ and MEG,^45^ we found the hemispheres were highly correlated in individuals with TLE across EEG and MEG, suggesting no lateralization value. Alpha slowing in MEG was significant for left and right TLE. Furthermore, we demonstrate the widespread spatial effect of slowing alpha rhythm (statistical results available in Supplementary Tables S9–S12).

The neurobiological explanation for the alpha rhythm slowing in epilepsy and other neurological conditions is unknown, although, large-scale networks are implicated.^24,46^ For example, alpha oscillations are closely related to corticothalamic networks,^1,2,8,9^ and are known to propagate towards the occipital areas.^2^ The thalamus is considered the peacemaker of alpha oscillations, having substantial control of the alpha rhythm.^1,2,9^ Alpha rhythm has distinct spatial and functional patterns, suggesting multiple alpha generators with separate roles.^7,9^ Specifically, the alpha rhythm is suspected to have divergent relationships with different parts of the default mode network.^47^

Sleep is a vital element of good health^15,48^ and neurophysiologically involves multiple networks, such as the default mode network.^49^ Sleep deprivation is associated with alpha rhythm alterations,^15^ reducing functional connectivity within the default mode network, and disrupted coupling within highly integrated and highly isolated networks.^15,50^ GABA neurotransmitters regulate sleep patterns and compromised function of GABAergic function impacts upon the quality of sleep.^51^ Furthermore, the correlation between sleep deprivation and exacerbation of epileptic seizures is well acknowledged,^24,52,53^ and GABAergic dysfunction is hypothesized to be a major mechanism.^54^ Similar findings have been reported in Alzheimer’s disease, regarding alpha rhythm alterations,^22^ interrupted sleep^55^ and disrupted GABA function.^22,32,34^ Collectively, a pathological link may exist between epilepsy and Alzheimer’s disease, presenting with similar symptoms and mechanisms.

This study had several strengths and limitations. One strength is the similarity of our results across two independent cohorts with different modalities, confirming published results. The subjects were approximately age- and sex-matched across both modalities. Additionally, eye-closure was ensured during data collection in scalp EEG and MEG. The limitations of this study include small-sample sizes that narrow the variability pool, and statistical significance should therefore be interpreted with caution across groups. Potential age effects on band power were not considered,^56-58^ as our sample consisted of a limited number of adult subjects only, precluding the construction of an accurate age-effect model. MEG data were collected at two different sites, which we did not control for. However, given that we were able to replicate previous results with data acquired in different sites, this suggests the alpha slowing effect is likely not driven by site differences. We also did not account for different epilepsy syndromes. The influence of daydreaming, circadian and ultradian rhythms on brain and bodily functions is widely acknowledged^59-61^; however, their effects on cerebral functions and power remain a challenging area of research and were beyond the scope of this study. Furthermore, antiseizure medication are strong modulators of band power,^26,36^ which was not addressed in this study. Future studies could investigate the relationship between alpha rhythm changes and antiseizure medication usage. Another potential limitation may be occasional interictal spikes that could contaminate the regional band power, although previous work suggests little or no effect on power spectral densities.^14^ Lastly, a recent study found that asymptomatic relatives of individuals with epilepsy, not taking medication, also had reduced alpha frequency peak in the posterior regions and compromised somatosensory network function.^26^ This suggests that alpha rhythm slowing may reflect a genetically determined tendency to have seizures.

In this study, we attempted to replicate the methods in Abela *et al.*^24^ and Kudo *et al.*^27^. We have found significant alpha slowing in individuals with TLE compared to controls, and a forward spread of the slow alpha, recorded with scalp EEG and MEG. Diverse neuropsychiatric diseases directly relate alpha rhythm alterations to behavioural symptoms. While we expand the epilepsy literature, we suggest that alpha slowing is a non-specific pathological symptom related to compromised neural networks, genetic mutations and abnormal GABA expression. Future studies could explore whether alpha rhythm slowing could be reversed in people with epilepsy, and the implications of this.

## Supplementary Material

fcae439_Supplementary_Data

## Data Availability

Data and code to reproduce the main findings of the study are available here: https://zenodo.org/records/13171578.
