# Supplementary material for: Alpha rhythm slowing in temporal lobe epilepsy across scalp EEG and MEG
Source: Brain Commun. 2024 Dec 5;6(6):fcae439. doi: 10.1093/braincomms/fcae439 (PMC11650000; doi:10.1093/braincomms/fcae439)
Supplement: fcae439_Supplementary_Data [file fcae439_supplementary_data.docx]

**Supplementary**

**Supplementary Material 1: Scalp EEG patient and healthy cohort metadata tables**

In the main manuscript we provided descriptive statistics of patients and healthy controls in terms of age, sex (M/F), lateralisation side (Left/Right), age of epilepsy onset and duration in Table 1, across scalp EEG and MEG cohorts. Here we provide more information on individual subject basis and channels that were removed in scalp EEG for individual subject during pre-processing. Note first pathology abbreviations: CAV (Cavernoma), HS (Hippocampal Sclerosis), EFS (End Folium Sclerosis). Note anti-seizure medications (ASM) abbreviations: CBZ (Carbamazepine), CLB (Clobazam), CLBprn (Clobazam, ‘pro re nata’), LCS (Lacosamide), LTG (Lamotrigine), LEV (Levetiracetam), OXC (Oxcarbazepine), PGB (Pregabalin), PHT (Phenytol),TMP (Trimethoprim), VPA (Valproic acid), ZNS (Zonisamide)

**Table S1: Summary of scalp EEG patient data**

| **Patient ID** | **Age** | **Age at Onset** | **Duration** | **Sex** | **Side** | **First Pathology** | **Anti-Seizure Medication** | **Channels Removed** |
| --- | --- | --- | --- | --- | --- | --- | --- | --- |
| 1 | 24 | 15 | 9 | F | Left | Other | LTG | - |
| 2 | 29 | 15 | 14 | F | Left | HS | LTG | - |
| 3 | 31 | 13 | 18 | F | Left | HS (EFS) | CBZ, LEV, CLBprn | - |
| 4 | 31 | 13 | 18 | F | Left | HS (EFS) | CBZ, LEV, CLBprn | - |
| 5 | 33 | 15 | 18 | M | Right | Other | CBZ, CLB, LTG, VPA | - |
| 6 | 19 | 7.5 | 11.5 | F | Left | HS | LTG, TMP | - |
| 7 | 42 | 3 | 39 | F | Left | Other | LEV, VPA, ZNS | TP10, O2 |
| 8 | 24 | 19 | 5 | M | Right | Other | CLB, VPA, PHT | - |
| 9 | 47 | 14 | 33 | M | Right | Other | CBZ, TMP | - |
| 10 | 25 | 9 | 16 | F | Left | HS | CLB, LEV, PHT, PGB | F7, F8 |
| 11 | 38 | 30 | 8 | F | Right | HS | LGT, LEV, VPA | - |
| 12 | 48 | 4 | 44 | M | Left | HS | CBZ, LEV, CLB | - |
| 13 | 47 | 9 | 38 | F | Left | HS | CBZ, LEV, PHT | - |
| 14 | 57 | 5 | 52 | M | Right | Other | CBZ, CLB | O1, O2, Oz |
| 15 | 33 | 2 | 31 | M | Left | HS | LEV, LTG | - |
| 16 | 26 | 7 | 19 | M | Left | HS | VPA, LCS | - |
| 17 | 35 | 18 | 17 | M | Right | Other | CBZ, LTG, LEV, ZNS, PHT | TP9 |
| 18 | 31 | 24 | 7 | F | Left | Other | LEV, PGB, VPA | - |
| 19 | 32 | 10 | 22 | M | Left | HS | LCS, LTG | Fp1, Fp2, F8 |
| 20 | 45 | 17 | 28 | F | Right | HS (EFS) | LEV, TMP | P7, C3 |
| 21 | 19 | 3 | 16 | F | Left | HS (EFS) | LEV, TMP | - |
| 22 | 37 | 32 | 5 | F | Right | Other | LEV, OCMZ | Fp2 |

**Table S2: Summary of scalp EEG healthy control data**

| **Control ID** | **Age** | **Sex** | **Channels Removed** |
| --- | --- | --- | --- |
| 1 | 28 | F | - |
| 2 | 39 | M | - |
| 3 | 49 | F | - |
| 4 | 29 | F | - |
| 5 | 41 | M | - |
| 6 | 35 | M | - |
| 7 | 28 | M | - |
| 8 | 30 | M | - |
| 9 | 30 | F | - |
| 10 | 33 | F | - |
| 11 | 28 | M | T7 |
| 12 | 22 | M | - |
| 13 | 30 | M | - |
| 14 | 28 | M | T8 |
| 15 | 34 | M | - |
| 16 | 25 | F | T8 |
| 17 | 33 | M | - |

**Table S3: Summary of MEG patient data**

| **Patient ID** | **Age** | **Age of Onset** | **Duration** | **Sex** | **Side** | **First pathology** | **Anti-Seizure Medication** |
| --- | --- | --- | --- | --- | --- | --- | --- |
| 1 | 38 | 32 | 6.3 | F | Right | HS | LEV, OXC |
| 2 | 29 | 6 | 22.7 | M | Left | OTHER | CBZ, LEV, LTG |
| 3 | 34 | 15 | 18.6 | M | Right | OTHER | CBZ, CLB, LTG, VPA |
| 4 | 32 | 24 | 7.6 | F | Left | OTHER | CBZ, LTG, LEV, ZNS, PHT |
| 5 | 24 | 15 | 8.6 | F | Left | OTHER | LTG |
| 6 | 30 | 10 | 19.8 | M | Left | HS(EFS) | LEV, PGB, VPA |
| 7 | 25 | 19 | 5.7 | M | Right | OTHER | CLB, VPA, PHT |
| 8 | 31 | 15 | 16.3 | M | Left | OTHER | CBZ, CLB,LEV |
| 9 | 42 | 10 | 31.9 | F | Right | OTHER | LTG, LEV |
| 10 | 48 | 14 | 33.7 | M | Right | OTHER | CBZ, TMP |
| 11 | 35 | 18 | 17 | M | Right | OTHER | VPA, LCS |
| 12 | 48 | 22 | 26 | F | Right | HS | PGB, OXC, TMP, CLB |
| 13 | 27 | 22 | 5.1 | F | Right | OTHER | LEV, LTG |
| 14 | 23 | 15 | 8.4 | F | Right | OTHER | OXC |
| 15 | 31 | 7 | 23.7 | F | Right | HS | CLB, LEV, PBG, VPA |
| 16 | 54 | 18 | 35.6 | F | Right | CAV | LEV, PGB |
| 17 | 25 | 0.66 | 24.34 | F | Right | HS | LEV, OXC |
| 18 | 53 | 0.01 | 52.99 | M | Left | HS | CLB, LCS, LEV, PGB |
| 19 | 26 | 13 | 13.3 | M | Right | OTHER | CBZ, CLB |
| 20 | 27 | 15 | 11.7 | M | Left | OTHER | CBZ, LCS |
| 21 | 21 | 11 | 9.6 | M | Right | HS | CLB, TMP, ZNS |
| 22 | 33 | 7 | 26 | M | Left | HS & OTHER | LTG, LEV, OXC |
| 23 | 38 | 6 | 32.4 | F | Left | HS | CLB, LTG, ZNS |
| 24 | 42 | 25 | 17 | F | Right | HS | LTG, CLB |

**Table S4: Summary of MEG healthy control data**

| **Control ID** | **Age** | **Sex** |
| --- | --- | --- |
| 1 | 36 | F |
| 2 | 44 | M |
| 3 | 32 | F |
| 4 | 55 | F |
| 5 | 27 | F |
| 6 | 31 | M |
| 7 | 23 | F |
| 8 | 30 | F |
| 9 | 26 | F |
| 10 | 28 | M |
| 11 | 23 | F |
| 12 | 35 | M |
| 13 | 37 | M |
| 14 | 27 | M |
| 15 | 32 | F |
| 16 | 23 | F |
| 17 | 30 | F |
| 18 | 25 | M |
| 19 | 25 | F |
| 20 | 27 | F |
| 21 | 32 | F |
| 22 | 39 | M |
| 23 | 32 | M |
| 24 | 45 | F |
| 25 | 37 | F |
| 26 | 36 | M |
| 27 | 28 | M |
| 28 | 26 | F |
| 29 | 33 | F |
| 30 | 28 | F |
| 31 | 24 | F |
| 32 | 32 | F |
| 33 | 25 | F |
| 34 | 26 | M |
| 35 | 25 | F |
| 36 | 26 | F |
| 37 | 31 | M |
| 38 | 27 | F |
| 39 | 26 | F |
| 40 | 27 | M |
| 41 | 26 | F |
| 42 | 31 | F |
| 43 | 34 | M |
| 44 | 24 | M |
| 45 | 41 | F |

**Supplementary Material 3: Scalp EEG and MEG t-test and Bonferroni correction result tables.** In the main manuscript Figure 3 illustrates the alpha rhythm ratio in individual healthy control and TLE patients, across scalp EEG and MEG modalities. Here we provide the unpaired t-test result between each hemisphere group. Significant p-values are indicated in bold. Note abbreviations: TLE (Temporal Lobe Epilepsy) and Hemi (hemisphere).

**Table S5: Scalp EEG t-test results**

|  |  | Control  (Left Hemi) | Control (Right Hemi) | Left TLE (Left Hemi) | Left TLE (Right Hemi) | Right TLE (Left Hemi) | Right TLE (Right Hemi) |
| --- | --- | --- | --- | --- | --- | --- | --- |
| **P-value** | Control (Left Hemi) | [] | 0.898 | **0.000** | **0.001** | 0.082 | 0.093 |
|  | Control (Right Hemi) | [] | [] | **0.000** | **0.001** | 0.066 | 0.075 |
|  | Left TLE (Left Hemi) | [] | [] | [] | 0.893 | 0.074 | 0.069 |
|  | Left TLE (Right Hemi) | [] | [] | [] | [] | 0.110 | 0.102 |
|  | Right TLE (Left Hemi) | [] | [] | [] | [] | [] | 0.948 |
|  | Right TLE (Right Hemi) | [] | [] | [] | [] | [] | [] |
|  |  |  |  |  |  |  |  |
| **Tstat** | Control (Left Hemi) | [] | 0.129 | -3.981 | -3.768 | -1.821 | -1.755 |
|  | Control (Right Hemi) | [] | [] | -4.107 | -3.891 | -1.931 | -1.865 |
|  | Left TLE (Left Hemi) | [] | [] | [] | 0.136 | 1.884 | 1.923 |
|  | Left TLE (Right Hemi) | [] | [] | [] | [] | 1.672 | 1.712 |
|  | Right TLE (Left Hemi) | [] | [] | [] | [] | [] | -0.066 |
|  | Right TLE (Right Hemi) | [] | [] | [] | [] | [] | [] |

**Table S6: Scalp EEG Bonferroni corrected t-test results**

|  |  |  | | | | | |
| --- | --- | --- | --- | --- | --- | --- | --- |
|  |  | Control (Left Hemi) | Control (Right Hemi) | Left TLE (Left Hemi) | Left TLE (Right Hemi) | Right TLE (Left Hemi) | Right TLE (Right Hemi) |
| **P-value** | Control (Left Hemi) | [] | 2.679 | **0.006** | **0.009** | 0.600 | 0.571 |
|  | Control (Right Hemi) | [] | [] | **0.004** | **0.007** | 0.725 | 0.667 |
|  | Left TLE (Left Hemi) | [] | [] | [] | 2.679 | 0.688 | 0.725 |
|  | Left TLE (Right Hemi) | [] | [] | [] | [] | 0.511 | 0.555 |
|  | Right TLE (Left Hemi) | [] | [] | [] | [] | [] | 1.797 |
|  | Right TLE (Right Hemi) | [] | [] | [] | [] | [] | [] |
|  |  |  |  |  |  |  |  |

**Table S7: MEG t-test results**

|  |  | Control  (Left Hemi) | Control (Right Hemi) | Left TLE (Left Hemi) | Left TLE (Right Hemi) | Right TLE (Left Hemi) | Right TLE (Right Hemi) |
| --- | --- | --- | --- | --- | --- | --- | --- |
| **P-value** | Control (Left Hemi) | [] | 0.948 | **0.000** | **0.000** | **0.000** | **0.000** |
|  | Control (Right Hemi) | [] | [] | **0.000** | **0.000** | **0.000** | **0.000** |
|  | Left TLE (Left Hemi) | [] | [] | [] | 0.911 | 0.850 | 0.834 |
|  | Left TLE (Right Hemi) | [] | [] | [] | [] | 0.937 | 0.923 |
|  | Right TLE (Left Hemi) | [] | [] | [] | [] | [] | 0.986 |
|  | Right TLE (Right Hemi) | [] | [] | [] | [] | [] | [] |
|  |  |  |  |  |  |  |  |
| **Tstat** | Control (Left Hemi) | [] | -0.065 | -4.449 | -4.556 | -5.625 | -5.674 |
|  | Control (Right Hemi) | [] | [] | -4.423 | -4.530 | -5.691 | -5.640 |
|  | Left TLE (Left Hemi) | [] | [] | [] | -0.113 | -0.192 | -0.212 |
|  | Left TLE (Right Hemi) | [] | [] | [] | [] | -0.081 | -0.098 |
|  | Right TLE (Left Hemi) | [] | [] | [] | [] | [] | 0.017 |
|  | Right TLE (Right Hemi) | [] | [] | [] | [] | [] | [] |

**Table S8: MEG Bonferroni corrected t-test results**

|  |  |  | | | | | |
| --- | --- | --- | --- | --- | --- | --- | --- |
|  |  | Control (Left Hemi) | Control (Right Hemi) | Left TLE (Left Hemi) | Left TLE (Right Hemi) | Right TLE (Left Hemi) | Right TLE (Right Hemi) |
| **P-value** | Control (Left Hemi) | [] | 2.810 | **0.000** | **0.000** | **0.000** | **0.000** |
|  | Control (Right Hemi) | [] | [] | **0.000** | **0.000** | **0.000** | **0.000** |
|  | Left TLE (Left Hemi) | [] | [] | [] | 5.098 | 5.839 | 5.839 |
|  | Left TLE (Right Hemi) | [] | [] | [] | [] | 3.690 | 4.557 |
|  | Right TLE (Left Hemi) | [] | [] | [] | [] | [] | 1.896 |
|  | Right TLE (Right Hemi) | [] | [] | [] | [] | [] | [] |
|  |  |  |  |  |  |  |  |

**Supplementary Material 4: Scalp EEG and MEG t-test and Bonferroni correction result tables.** In the main manuscript Figure 4 illustrates the widespread effect of alpha rhythm ratio, across scalp EEG and MEG modalities. Here we provide the paired t-test result between each hemisphere group. Significant p-values are indicated in bold. Note abbreviations: TLE (Temporal Lobe Epilepsy) and Hemi (hemisphere).

**Table S9: Scalp EEG t-test results**

|  |  | Control  (Left Hemi) | Control (Right Hemi) | Left TLE (Left Hemi) | Left TLE (Right Hemi) | Right TLE (Left Hemi) | Right TLE (Right Hemi) |
| --- | --- | --- | --- | --- | --- | --- | --- |
| **P-value** | Control (Left Hemi) | [] | **0.006** | **0.000** | **0.000** | **0.000** | **0.000** |
|  | Control (Right Hemi) | [] | [] | **0.000** | **0.000** | **0.000** | **0.000** |
|  | Left TLE (Left Hemi) | [] | [] | [] | **0.012** | **0.000** | **0.000** |
|  | Left TLE (Right Hemi) | [] | [] | [] | [] | **0.000** | **0.000** |
|  | Right TLE (Left Hemi) | [] | [] | [] | [] | [] | 0.231 |
|  | Right TLE (Right Hemi) | [] | [] | [] | [] | [] | [] |
|  |  |  |  |  |  |  |  |
| **Tstat** | Control (Left Hemi) | [] | 2.930 | -54.415 | -56.053 | -16.956 | -18.280 |
|  | Control (Right Hemi) | [] | [] | -53.656 | -59.901 | -16.642 | -17.753 |
|  | Left TLE (Left Hemi) | [] | [] | [] | 2.665 | 20.428 | 23.068 |
|  | Left TLE (Right Hemi) | [] | [] | [] | [] | 16.441 | 21.524 |
|  | Right TLE (Left Hemi) | [] | [] | [] | [] | [] | -1.219 |
|  | Right TLE (Right Hemi) | [] | [] | [] | [] | [] | [] |

**Table S10: Scalp EEG Bonferroni corrected t-test results**

|  |  |  | | | | | |
| --- | --- | --- | --- | --- | --- | --- | --- |
|  |  | Control (Left Hemi) | Control (Right Hemi) | Left TLE (Left Hemi) | Left TLE (Right Hemi) | Right TLE (Left Hemi) | Right TLE (Right Hemi) |
| **P-value** | Control (Left Hemi) | [] | **0.018** | **0.000** | **0.000** | **0.000** | **0.000** |
|  | Control (Right Hemi) | [] | [] | **0.000** | **0.000** | **0.000** | **0.000** |
|  | Left TLE (Left Hemi) | [] | [] | [] | **0.024** | **0.000** | **0.000** |
|  | Left TLE (Right Hemi) | [] | [] | [] | [] | **0.000** | **0.000** |
|  | Right TLE (Left Hemi) | [] | [] | [] | [] | [] | 0.231 |
|  | Right TLE (Right Hemi) | [] | [] | [] | [] | [] | [] |
|  |  |  |  |  |  |  |  |

**Table S11: MEG t-test results**

|  |  | Control  (Left Hemi) | Control (Right Hemi) | Left TLE (Left Hemi) | Left TLE (Right Hemi) | Right TLE (Left Hemi) | Right TLE (Right Hemi) |
| --- | --- | --- | --- | --- | --- | --- | --- |
| **P-value** | Control (Left Hemi) | [] | 0.413 | **0.000** | **0.000** | **0.000** | **0.000** |
|  | Control (Right Hemi) | [] | [] | **0.000** | **0.000** | **0.000** | **0.000** |
|  | Left TLE (Left Hemi) | [] | [] | [] | 0.134 | **0.007** | **0.011** |
|  | Left TLE (Right Hemi) | [] | [] | [] | [] | 0.253 | 0.188 |
|  | Right TLE (Left Hemi) | [] | [] | [] | [] | [] | 0.718 |
|  | Right TLE (Right Hemi) | [] | [] | [] | [] | [] | [] |
|  |  |  |  |  |  |  |  |
| **Tstat** | Control (Left Hemi) | [] | -0.830 | -20.905 | -20.577 | -23.104 | -22.051 |
|  | Control (Right Hemi) | [] | [] | -22.349 | -21.154 | -23.870 | -22.677 |
|  | Left TLE (Left Hemi) | [] | [] | [] | -1.535 | -2.693 | -2.891 |
|  | Left TLE (Right Hemi) | [] | [] | [] | [] | -1.640 | -1.344 |
|  | Right TLE (Left Hemi) | [] | [] | [] | [] | [] | 0.364 |
|  | Right TLE (Right Hemi) | [] | [] | [] | [] | [] | [] |

**Table S12: MEG Bonferroni corrected t-test results**

|  |  |  | | | | | |
| --- | --- | --- | --- | --- | --- | --- | --- |
|  |  | Control (Left Hemi) | Control (Right Hemi) | Left TLE (Left Hemi) | Left TLE (Right Hemi) | Right TLE (Left Hemi) | Right TLE (Right Hemi) |
| **P-value** | Control (Left Hemi) | [] | 0.825 | **0.000** | **0.000** | **0.000** | **0.000** |
|  | Control (Right Hemi) | [] | [] | **0.000** | **0.000** | **0.000** | **0.000** |
|  | Left TLE (Left Hemi) | [] | [] | [] | 0.671 | **0.047** | **0.066** |
|  | Left TLE (Right Hemi) | [] | [] | [] | [] | 0.758 | 0.753 |
|  | Right TLE (Left Hemi) | [] | [] | [] | [] | [] | 0.825 |
|  | Right TLE (Right Hemi) | [] | [] | [] | [] | [] | [] |
|  |  |  |  |  |  |  |  |

**Supplementary Material 5: Alpha power ratio matrices by regions of interest in Scalp EEG and MEG.** In the main manuscript Figure 4 illustrates the widespread effect of maximum alpha power ratio, across scalp EEG and MEG modalities. Here we provide the full alpha power ratio matrices used to produce the brain plots.

|  | **EEG Controls** | | **EEG left TLE** | | **EEG right TLE** | |
| --- | --- | --- | --- | --- | --- | --- |
|  | left hemisphere | right hemisphere | left hemisphere | right hemisphere | left hemisphere | right hemisphere |
| bankssts | -0.2354 | -0.2538 | 0.5072 | 0.4130 | 0.2011 | 0.0911 |
| caudal anterior cingulate | -0.2943 | -0.2784 | 0.4091 | 0.3796 | 0.2530 | 0.1883 |
| caudal middle frontal | -0.1115 | -0.1104 | 0.4864 | 0.4256 | 0.1715 | 0.0656 |
| cuneus | -0.4181 | -0.3730 | 0.3325 | 0.3250 | 0.1762 | 0.1682 |
| entorhinal | -0.1423 | -0.1491 | 0.5728 | 0.5776 | 0.1349 | 0.1359 |
| fusiform | -0.1404 | -0.1205 | 0.5997 | 0.5585 | 0.0942 | 0.1281 |
| inferior parietal | -0.3986 | -0.4565 | 0.3805 | 0.2852 | 0.1540 | 0.0982 |
| inferior temporal | -0.1227 | -0.1138 | 0.4681 | 0.5052 | 0.1863 | 0.1752 |
| isthmus cingulate | -0.2853 | -0.2646 | 0.4273 | 0.3594 | 0.1587 | 0.0771 |
| lateral occipital | -0.3590 | -0.4862 | 0.3484 | 0.3109 | 0.1567 | 0.0931 |
| lateral orbitofrontal | -0.2279 | -0.2325 | 0.4606 | 0.4825 | 0.1071 | 0.1880 |
| lingual | -0.3190 | -0.3273 | 0.3727 | 0.4638 | 0.0748 | 0.1616 |
| medial orbitofrontal | -0.1267 | -0.1943 | 0.5194 | 0.4772 | 0.1375 | 0.1656 |
| middle temporal | -0.0887 | -0.1024 | 0.5121 | 0.4929 | 0.1130 | 0.1478 |
| parahippocampal | -0.1376 | -0.1628 | 0.4626 | 0.5188 | 0.1722 | 0.1506 |
| paracentral | -0.1692 | -0.2096 | 0.3861 | 0.3765 | 0.0835 | 0.0638 |
| pars opercularis | -0.1638 | -0.2734 | 0.4430 | 0.4216 | 0.1120 | 0.1370 |
| pars orbitalis | -0.2328 | -0.2342 | 0.5064 | 0.4688 | 0.1128 | 0.1105 |
| pars triangularis | -0.0862 | -0.1533 | 0.4027 | 0.4517 | 0.1871 | 0.2084 |
| pericalcarine | -0.3901 | -0.4089 | 0.3754 | 0.3438 | 0.0614 | 0.1112 |
| postcentral | -0.2349 | -0.2513 | 0.4145 | 0.3497 | 0.0713 | 0.0307 |
| posterior cingulate | -0.1548 | -0.1813 | 0.4615 | 0.4778 | 0.1923 | 0.2211 |
| precentral | -0.2193 | -0.2174 | 0.4180 | 0.3970 | 0.0773 | 0.0397 |
| precuneus | -0.3887 | -0.4274 | 0.3503 | 0.3386 | 0.1978 | 0.1158 |
| rostral anterior cingulate | -0.1095 | -0.1872 | 0.4942 | 0.4063 | 0.1368 | 0.1336 |
| rostral middle frontal | -0.1149 | -0.2064 | 0.4844 | 0.4752 | 0.2224 | 0.2490 |
| superior frontal | -0.0750 | -0.0826 | 0.4739 | 0.4558 | 0.0816 | 0.0998 |
| superior parietal | -0.4185 | -0.4907 | 0.3044 | 0.2183 | 0.1891 | 0.1400 |
| superior temporal | -0.1456 | -0.1677 | 0.5071 | 0.5181 | 0.0984 | 0.1443 |
| supramarginal | -0.2839 | -0.3004 | 0.4549 | 0.3488 | 0.1054 | 0.0764 |
| frontal pole | -0.2066 | -0.1711 | 0.4962 | 0.4668 | 0.1587 | 0.1632 |
| temporal pole | -0.1406 | -0.0545 | 0.5103 | 0.5574 | 0.1202 | 0.1397 |
| transverse temporal | -0.1995 | -0.2006 | 0.4769 | 0.4298 | 0.1221 | 0.0694 |
| insula | -0.0744 | -0.1295 | 0.5288 | 0.5410 | 0.1502 | 0.1337 |

**S13: Scalp EEG alpha power ratio data across 34 regions of interest.**

**S14: MEG alpha power ratio data across 34 regions of interest.**

|  | **MEG Controls** | | **MEG left TLE** | | **MEG right TLE** | |
| --- | --- | --- | --- | --- | --- | --- |
|  | left hemisphere | right hemisphere | left hemisphere | right hemisphere | left hemisphere | right hemisphere |
| bankssts | -0.1447 | -0.1830 | 0.2414 | 0.3620 | 0.3417 | 0.3057 |
| caudal anterior cingulate | -0.1900 | -0.1898 | 0.2776 | 0.2772 | 0.2729 | 0.2989 |
| caudal middle frontal | -0.0873 | -0.0564 | 0.3400 | 0.3104 | 0.2721 | 0.3224 |
| cuneus | -0.4543 | -0.4301 | 0.2433 | 0.2071 | 0.2188 | 0.2057 |
| entorhinal | -0.1604 | -0.1838 | 0.2804 | 0.3530 | 0.3657 | 0.3649 |
| fusiform | -0.2098 | -0.1880 | 0.3207 | 0.2704 | 0.3843 | 0.3702 |
| inferior parietal | -0.3730 | -0.3879 | 0.1720 | 0.2685 | 0.2622 | 0.3035 |
| inferior temporal | -0.1389 | -0.1473 | 0.3380 | 0.3814 | 0.4068 | 0.4054 |
| isthmus cingulate | -0.2288 | -0.2356 | 0.2413 | 0.1836 | 0.2718 | 0.3136 |
| lateral occipital | -0.3171 | -0.3639 | 0.1796 | 0.2340 | 0.2360 | 0.2419 |
| lateral orbitofrontal | -0.1361 | -0.1252 | 0.3525 | 0.3516 | 0.3334 | 0.3494 |
| lingual | -0.3635 | -0.3331 | 0.2770 | 0.2832 | 0.2888 | 0.2791 |
| medial orbitofrontal | -0.0044 | -0.0170 | 0.3146 | 0.3078 | 0.3708 | 0.3912 |
| middle temporal | -0.1354 | -0.1603 | 0.3468 | 0.3386 | 0.3708 | 0.3985 |
| parahippocampal | -0.1658 | -0.1754 | 0.2759 | 0.3099 | 0.3228 | 0.3434 |
| paracentral | -0.2408 | -0.1531 | 0.2397 | 0.2377 | 0.2370 | 0.2600 |
| pars opercularis | -0.0636 | -0.1052 | 0.3484 | 0.3763 | 0.3272 | 0.3148 |
| pars orbitalis | 0.0230 | 0.0436 | 0.3416 | 0.3783 | 0.3595 | 0.3473 |
| pars triangularis | 0.0253 | 0.0251 | 0.3432 | 0.3512 | 0.3417 | 0.3268 |
| pericalcarine | -0.4287 | -0.3952 | 0.2760 | 0.2737 | 0.2192 | 0.2347 |
| postcentral | -0.2969 | -0.2692 | 0.2884 | 0.2430 | 0.2600 | 0.2538 |
| posterior cingulate | -0.1186 | -0.1244 | 0.2645 | 0.2789 | 0.2737 | 0.2700 |
| precentral | -0.2788 | -0.2338 | 0.2992 | 0.2110 | 0.2652 | 0.2413 |
| precuneus | -0.5144 | -0.4710 | 0.2273 | 0.2859 | 0.2193 | 0.2270 |
| rostral anterior cingulate | -0.0178 | -0.0015 | 0.3035 | 0.2679 | 0.3402 | 0.3338 |
| rostral middle frontal | 0.0667 | 0.0323 | 0.3020 | 0.3079 | 0.3124 | 0.2960 |
| superior frontal | -0.1789 | -0.1612 | 0.2355 | 0.2129 | 0.2505 | 0.2212 |
| superior parietal | -0.4024 | -0.4188 | 0.1864 | 0.2033 | 0.2555 | 0.2657 |
| superior temporal | -0.1749 | -0.1929 | 0.3504 | 0.4047 | 0.3611 | 0.4073 |
| supramarginal | -0.2685 | -0.2669 | 0.2568 | 0.3211 | 0.2810 | 0.2819 |
| frontal pole | 0.1221 | 0.0967 | 0.2416 | 0.2420 | 0.3151 | 0.2749 |
| temporal pole | -0.1426 | -0.1133 | 0.3190 | 0.3751 | 0.4178 | 0.3424 |
| transverse temporal | -0.1900 | -0.1786 | 0.4108 | 0.4139 | 0.3906 | 0.3756 |
| insula | -0.1831 | -0.1677 | 0.2952 | 0.3096 | 0.2804 | 0.3161 |

**Supplementary Material 6: Relative power ratio between slow and fast frequencies.** In the main manuscript, we use slow (6-9Hz) and fast (10-11Hz) alpha to illustrate the slowing of alpha band in individuals with TLE and spatially across the cortex.

Here, we combine delta and theta bands (1-7.5Hz) to represent slow frequency, and alpha and beta bands (8-30Hz) to represent fast frequency. We have replicated the main figures in the manuscript using the new slow and fast frequency data. Here, we also provide the unpaired and paired t-test results between each hemisphere group. Significant p-values are indicated in bold. Note abbreviations: TLE (Temporal Lobe Epilepsy) and Hemi (hemisphere).

We find that there is significantly more slow frequency in individuals with TLE compared to heathy control. We also see max relative power bilaterally dominant in the frontal regions across all groups with a greater effect seen in the patient cohorts (Fig. S2).


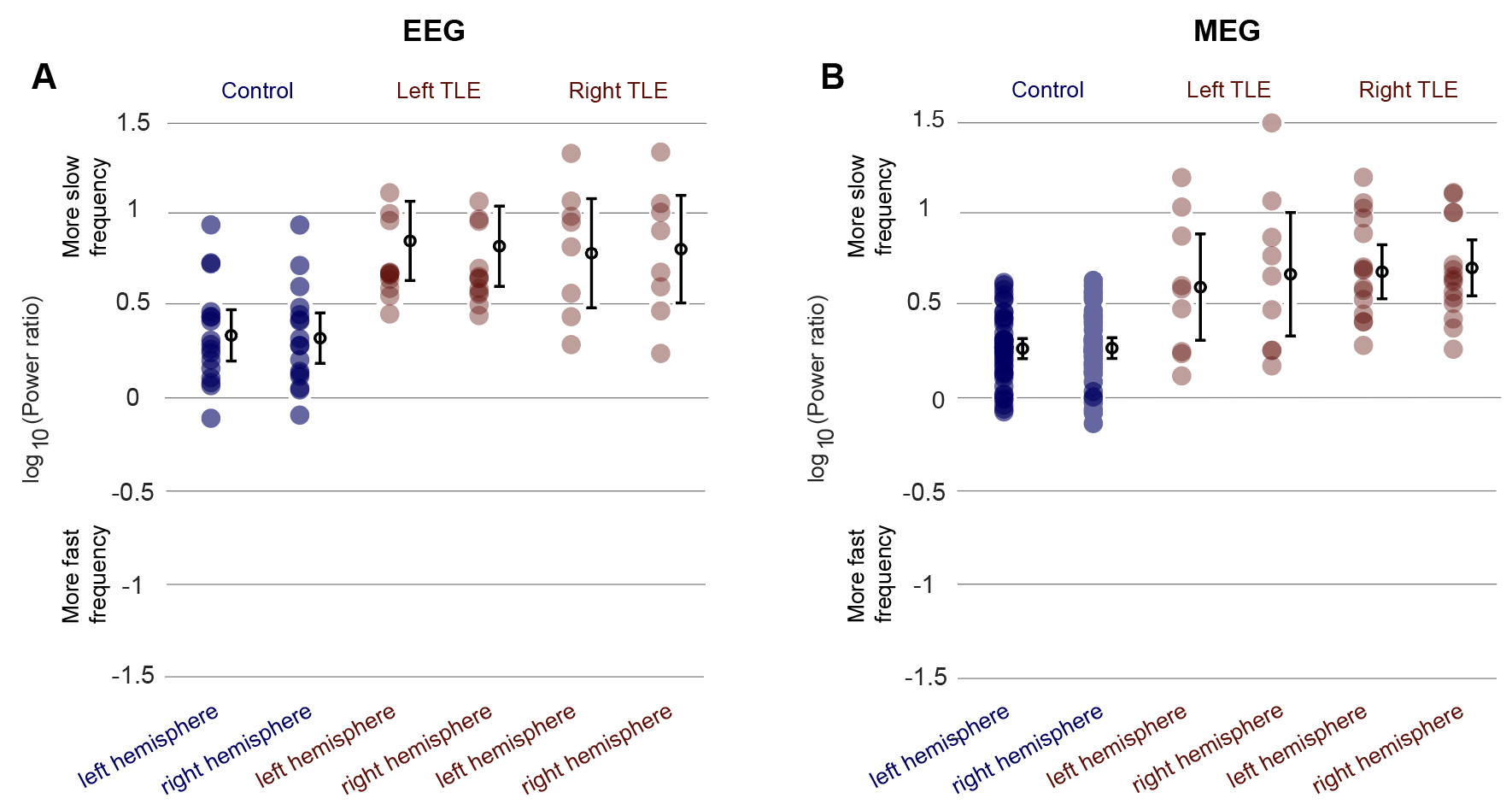


**Figure S1: Slow and fast frequency ratio in individual subjects in EEG vs MEG**. Illustrating the difference in slow-fast frequency ratio between individual healthy controls and individuals with left and right TLE, averaged across regions within a single hemisphere. Dots and error bars indicate 95% CI. Note the sample sizes for healthy control, left and right TLE patient cohorts in scalp EEG and (MEG): 17 (46), 14 (9) and 8 (15) (per hemisphere).

**Table S15: Scalp EEG t-test results**

|  |  | Control  (Left Hemi) | Control (Right Hemi) | Left TLE (Left Hemi) | Left TLE (Right Hemi) | Right TLE (Left Hemi) | Right TLE (Right Hemi) |
| --- | --- | --- | --- | --- | --- | --- | --- |
| **P-value** | Control (Left Hemi) | [] | 0.889 | **0.000** | **0.000** | **0.001** | **0.002** |
|  | Control (Right Hemi) | [] | [] | **0.000** | **0.000** | **0.001** | **0.001** |
|  | Left TLE (Left Hemi) | [] | [] | [] | 0.842 | 0.776 | 0.685 |
|  | Left TLE (Right Hemi) | [] | [] | [] | [] | 0.913 | 0.817 |
|  | Right TLE (Left Hemi) | [] | [] | [] | [] | [] | 0.910 |
|  | Right TLE (Right Hemi) | [] | [] | [] | [] | [] | [] |
|  |  |  |  |  |  |  |  |
| **Tstat** | Control (Left Hemi) | [] | 0.140 | -4.435 | -4.157 | -3.663 | -3.483 |
|  | Control (Right Hemi) | [] | [] | -4.579 | -4.298 | -3.804 | -3.621 |
|  | Left TLE (Left Hemi) | [] | [] | [] | 0.202 | 0.288 | 0.412 |
|  | Left TLE (Right Hemi) | [] | [] | [] | [] | 0.110 | 0.234 |
|  | Right TLE (Left Hemi) | [] | [] | [] | [] | [] | 0.115 |
|  | Right TLE (Right Hemi) | [] | [] | [] | [] | [] | [] |

**Table S16: Scalp EEG Bonferroni corrected t-test results**

|  |  |  | | | | | |
| --- | --- | --- | --- | --- | --- | --- | --- |
|  |  | Control (Left Hemi) | Control (Right Hemi) | Left TLE (Left Hemi) | Left TLE (Right Hemi) | Right TLE (Left Hemi) | Right TLE (Right Hemi) |
| **P-value** | Control (Left Hemi) | [] | 3.367 | **0.002** | **0.003** | **0.013** | **0.016** |
|  | Control (Right Hemi) | [] | [] | **0.001** | **0.002** | **0.010** | **0.013** |
|  | Left TLE (Left Hemi) | [] | [] | [] | 4.087 | 4.794 | 4.794 |
|  | Left TLE (Right Hemi) | [] | [] | [] | [] | 1.820 | 4.657 |
|  | Right TLE (Left Hemi) | [] | [] | [] | [] | [] | 2.668 |
|  | Right TLE (Right Hemi) | [] | [] | [] | [] | [] | [] |
|  |  |  |  |  |  |  |  |

**Table S17: MEG t-test results**

|  |  | Control  (Left Hemi) | Control (Right Hemi) | Left TLE (Left Hemi) | Left TLE (Right Hemi) | Right TLE (Left Hemi) | Right TLE (Right Hemi) |
| --- | --- | --- | --- | --- | --- | --- | --- |
| **P-value** | Control (Left Hemi) | [] | 0.948 | **0.000** | **0.000** | **0.000** | **0.000** |
|  | Control (Right Hemi) | [] | [] | **0.000** | **0.000** | **0.000** | **0.000** |
|  | Left TLE (Left Hemi) | [] | [] | [] | 0.720 | 0.530 | 0.450 |
|  | Left TLE (Right Hemi) | [] | [] | [] | [] | 0.926 | 0.826 |
|  | Right TLE (Left Hemi) | [] | [] | [] | [] | [] | 0.850 |
|  | Right TLE (Right Hemi) | [] | [] | [] | [] | [] | [] |
|  |  |  |  |  |  |  |  |
| **Tstat** | Control (Left Hemi) | [] | -0.065 | -4.090 | -4.617 | -6.797 | -7.004 |
|  | Control (Right Hemi) | [] | [] | -3.987 | -4.518 | -6.630 | -6.838 |
|  | Left TLE (Left Hemi) | [] | [] | [] | -0.365 | -0.638 | -0.769 |
|  | Left TLE (Right Hemi) | [] | [] | [] | [] | -0.094 | -0.223 |
|  | Right TLE (Left Hemi) | [] | [] | [] | [] | [] | -0.191 |
|  | Right TLE (Right Hemi) | [] | [] | [] | [] | [] | [] |

**Table S18: MEG Bonferroni corrected t-test results**

|  |  |  | | | | | |
| --- | --- | --- | --- | --- | --- | --- | --- |
|  |  | Control (Left Hemi) | Control (Right Hemi) | Left TLE (Left Hemi) | Left TLE (Right Hemi) | Right TLE (Left Hemi) | Right TLE (Right Hemi) |
| **P-value** | Control (Left Hemi) | [] | 1.853 | **0.001** | **0.000** | **0.000** | **0.000** |
|  | Control (Right Hemi) | [] | [] | **0.002** | **0.000** | **0.000** | **0.000** |
|  | Left TLE (Left Hemi) | [] | [] | [] | 3.599 | 3.180 | 3.150 |
|  | Left TLE (Right Hemi) | [] | [] | [] | [] | 3.550 | 3.600 |
|  | Right TLE (Left Hemi) | [] | [] | [] | [] | [] | 3.303 |
|  | Right TLE (Right Hemi) | [] | [] | [] | [] | [] | [] |
|  |  |  |  |  |  |  |  |

**
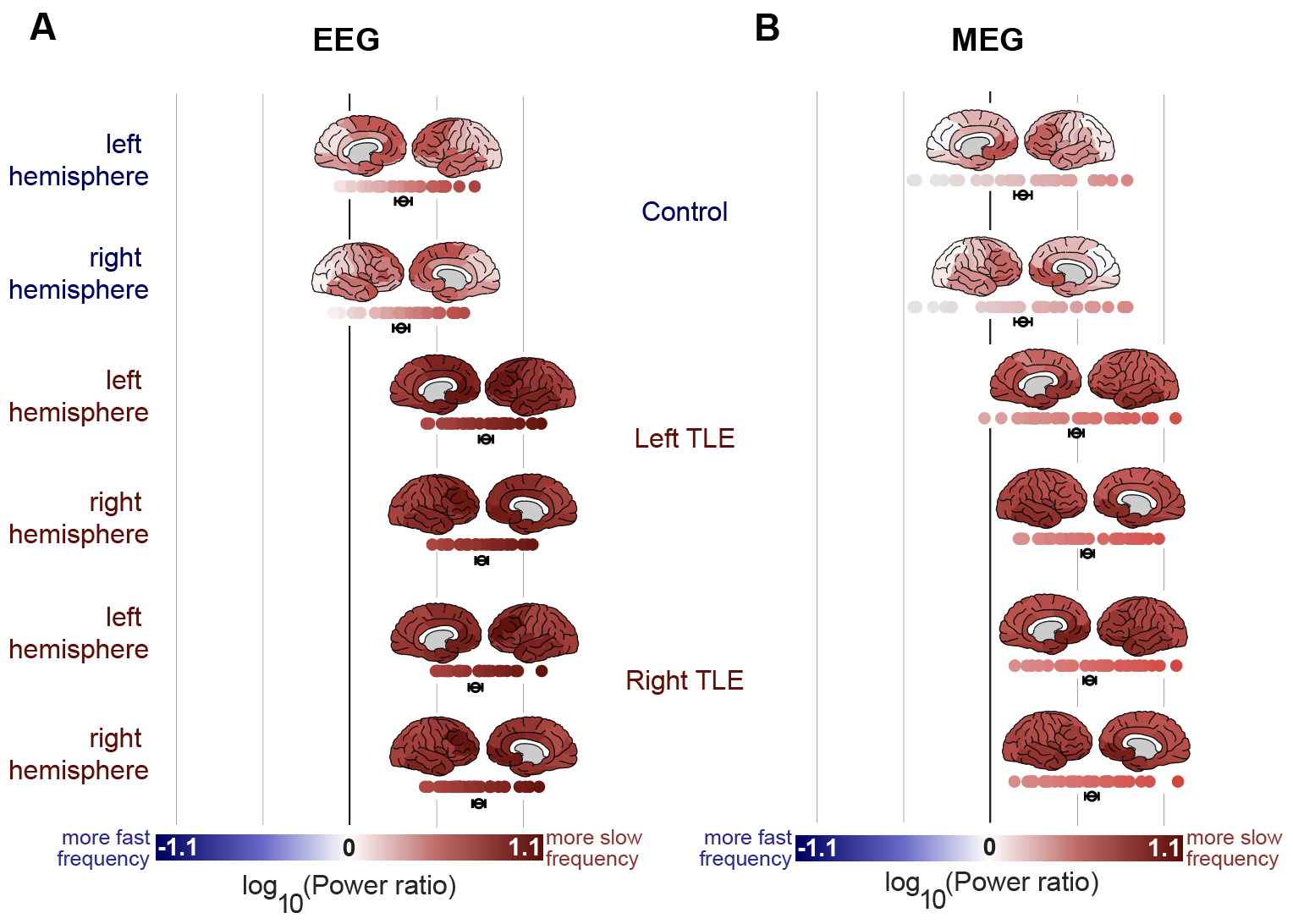
**

**Figure S2: Slow and fast frequency is spatially global across patients.** Frequency power ratio varies spatially across the cortex in left and right TLE patients compared to healthy controls. Dots and error bars indicate 95% CI. (A and B) There is more fast frequency in the healthy control cohort compared to individuals with TLE, in EEG and MEG. Slow frequency is maximal anteriorly and in the temporal regions, all cohort groups, in both modalities.

**Table S19: Scalp EEG t-test results**

|  |  | Control  (Left Hemi) | Control (Right Hemi) | Left TLE (Left Hemi) | Left TLE (Right Hemi) | Right TLE (Left Hemi) | Right TLE (Right Hemi) |
| --- | --- | --- | --- | --- | --- | --- | --- |
| **P-value** | Control (Left Hemi) | [] | 0.253 | **0.000** | **0.000** | **0.000** | **0.000** |
|  | Control (Right Hemi) | [] | [] | **0.000** | **0.000** | **0.000** | **0.000** |
|  | Left TLE (Left Hemi) | [] | [] | [] | **0.011** | **0.000** | **0.000** |
|  | Left TLE (Right Hemi) | [] | [] | [] | [] | 0.093 | **0.000** |
|  | Right TLE (Left Hemi) | [] | [] | [] | [] | [] | 0.106 |
|  | Right TLE (Right Hemi) | [] | [] | [] | [] | [] | [] |
|  |  |  |  |  |  |  |  |
| **Tstat** | Control (Left Hemi) | [] | 1.163 | -45.132 | -35.166 | -35.075 | -25.111 |
|  | Control (Right Hemi) | [] | [] | -35.556 | -37.416 | -28.524 | -25.021 |
|  | Left TLE (Left Hemi) | [] | [] | [] | 2.678 | 4.507 | 5.032 |
|  | Left TLE (Right Hemi) | [] | [] | [] | [] | 1.731 | 3.981 |
|  | Right TLE (Left Hemi) | [] | [] | [] | [] | [] | 1.662 |
|  | Right TLE (Right Hemi) | [] | [] | [] | [] | [] | [] |

**Table S20: Scalp EEG Bonferroni corrected t-test results**

|  |  |  | | | | | |
| --- | --- | --- | --- | --- | --- | --- | --- |
|  |  | Control (Left Hemi) | Control (Right Hemi) | Left TLE (Left Hemi) | Left TLE (Right Hemi) | Right TLE (Left Hemi) | Right TLE (Right Hemi) |
| **P-value** | Control (Left Hemi) | [] | 0.253 | **0.000** | **0.000** | **0.000** | **0.000** |
|  | Control (Right Hemi) | [] | [] | **0.000** | **0.000** | **0.000** | **0.000** |
|  | Left TLE (Left Hemi) | [] | [] | [] | **0.046** | **0.000** | **0.000** |
|  | Left TLE (Right Hemi) | [] | [] | [] | [] | 0.279 | **0.002** |
|  | Right TLE (Left Hemi) | [] | [] | [] | [] | [] | 0.279 |
|  | Right TLE (Right Hemi) | [] | [] | [] | [] | [] | [] |
|  |  |  |  |  |  |  |  |

**Table S21: MEG t-test results**

|  |  | Control  (Left Hemi) | Control (Right Hemi) | Left TLE (Left Hemi) | Left TLE (Right Hemi) | Right TLE (Left Hemi) | Right TLE (Right Hemi) |
| --- | --- | --- | --- | --- | --- | --- | --- |
| **P-value** | Control (Left Hemi) | [] | 0.346 | **0.000** | **0.000** | **0.000** | **0.000** |
|  | Control (Right Hemi) | [] | [] | **0.000** | **0.000** | **0.000** | **0.000** |
|  | Left TLE (Left Hemi) | [] | [] | [] | **0.000** | **0.000** | **0.000** |
|  | Left TLE (Right Hemi) | [] | [] | [] | [] | 0.282 | **0.033** |
|  | Right TLE (Left Hemi) | [] | [] | [] | [] | [] | **0.011** |
|  | Right TLE (Right Hemi) | [] | [] | [] | [] | [] | [] |
|  |  |  |  |  |  |  |  |
| **Tstat** | Control (Left Hemi) | [] | -0.956 | -14.388 | -16.339 | -25.162 | -33.983 |
|  | Control (Right Hemi) | [] | [] | -14.483 | -16.332 | -25.428 | -34.211 |
|  | Left TLE (Left Hemi) | [] | [] | [] | -5.771 | -7.037 | -7.278 |
|  | Left TLE (Right Hemi) | [] | [] | [] | [] | -1.093 | -2.230 |
|  | Right TLE (Left Hemi) | [] | [] | [] | [] | [] | -2.682 |
|  | Right TLE (Right Hemi) | [] | [] | [] | [] | [] | [] |

**Table S22: MEG Bonferroni corrected t-test results**

|  |  |  | | | | | |
| --- | --- | --- | --- | --- | --- | --- | --- |
|  |  | Control (Left Hemi) | Control (Right Hemi) | Left TLE (Left Hemi) | Left TLE (Right Hemi) | Right TLE (Left Hemi) | Right TLE (Right Hemi) |
| **P-value** | Control (Left Hemi) | [] | 0.564 | **0.000** | **0.000** | **0.000** | **0.000** |
|  | Control (Right Hemi) | [] | [] | **0.000** | **0.000** | **0.000** | **0.000** |
|  | Left TLE (Left Hemi) | [] | [] | [] | **0.000** | **0.000** | **0.000** |
|  | Left TLE (Right Hemi) | [] | [] | [] | [] | 0.564 | 0.098 |
|  | Right TLE (Left Hemi) | [] | [] | [] | [] | [] | **0.045** |
|  | Right TLE (Right Hemi) | [] | [] | [] | [] | [] | [] |
|  |  |  |  |  |  |  |  |

**Supplementary Material 7: effect of medication**

Here, we investigate the effects of taking particular ASM vs patients who do not take the ASM. In EEG, we looked at the individual effects of taking Carbamazepine (CBZ), Lamotrigine (LTG) and Levetiracetam (LEV) compared to patients who do not. Similarly in MEG, we looked at the individual ASM effects of Lamotrigine (LTG), Clobazam (CLB) and Levetiracetam (LEV). Overall, we found no significance, indicating that the ASM tested did not bias our results by affecting the alpha power ratio.

We note that small subject numbers between subgroups are an important limitation, and we cannot rule out the possibility ASM may have an effect on alpha power ratio in larger populations and taken as monotherapy medication.


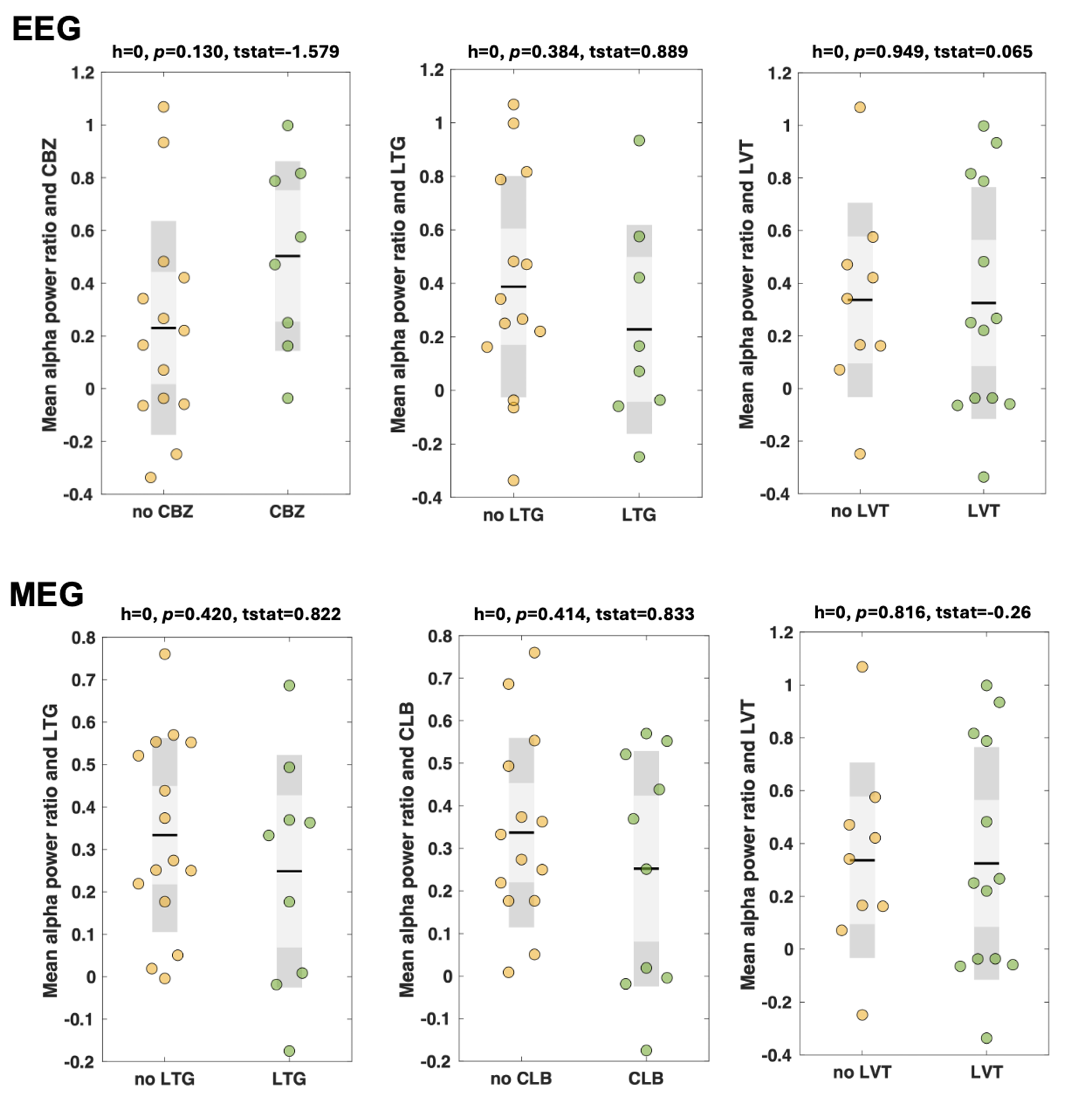


**Figure S3: Univariate scatterplots comparing individual ASM drugs.** In this analysis we investigated whether a particular antiseizure medication (ASM) can affect the mean power alpha ratio in individual patients.
